# Cognitive and Global Functioning in Patients With First-Episode Psychosis Stratified by Level of Negative Symptoms. A 10-Year Follow-Up Study

**DOI:** 10.3389/fpsyt.2022.841057

**Published:** 2022-03-25

**Authors:** Magnus Johan Engen, Anja Vaskinn, Ingrid Melle, Ann Færden, Siv Hege Lyngstad, Camilla Bärthel Flaaten, Line Hustad Widing, Kristin Fjelnseth Wold, Gina Åsbø, Beathe Haatveit, Carmen Simonsen, Torill Ueland

**Affiliations:** ^1^Division of Mental Health and Addiction, Nydalen DPS, Oslo University Hospital, Oslo, Norway; ^2^Department of Psychology, Faculty of Social Sciences, University of Oslo, Oslo, Norway; ^3^Norwegian Centre for Mental Disorders Research (NORMENT), Institute of Clinical Medicine, University of Oslo, Oslo, Norway; ^4^Centre for Research and Education in Forensic Psychiatry, Oslo University Hospital, Oslo, Norway; ^5^Division of Mental Health and Addiction, Section for Psychosis Research, Oslo University Hospital, Oslo, Norway; ^6^Division of Mental Health and Addiction, Department of Acute Psychiatry, Oslo University Hospital, Oslo, Norway; ^7^Early Intervention in Psychosis Advisory Unit for South-East Norway, Division of Mental Health and Addiction, Oslo University Hospital, Oslo, Norway

**Keywords:** cognition, longitudinal, negative symptoms (schizophrenia), attention, processing speed, executive functions, global functioning

## Abstract

Negative and cognitive symptoms are core features of schizophrenia that are correlated in cross-sectional designs. To further explore the relationship between these critical symptom dimensions we use a method for stratifying participants based on level and persistence of negative symptoms from absent to sustained levels over a 10-year follow-up period. We investigate associations with cognitive performance and level of global functioning. First-episode psychosis (FEP) participants (*n* = 102) and healthy controls (*n* = 116) were assessed at baseline and follow-up. A cognitive battery consisting of 14 tests derived into four domains and a composite score were used in the analyses. FEP participants were stratified based on negative symptom items from the Positive and Negative Syndrome Scale (PANSS-R) into four groups with either no, mild, transitory or sustained symptoms over the 10-year follow-up period. Global functioning was measured with Global Assessment of Functioning Scale-Split version. Multivariate and univariate analyses of variance were used to explore between-group differences in level and course of cognitive performance as global functioning. A multivariate analysis with four cognitive domains as dependent variables, showed significant group differences in performance when including healthy controls and the negative symptom groups. The groups with no and mild negative symptoms outperformed the group with sustained levels of negative symptoms on verbal learning and memory. The group with no negative symptoms also outperformed the group with sustained negative symptoms on the cognitive composite score. Significant improvements on verbal learning and memory, executive functioning and the cognitive composite were detected for the entire sample. No differences in cognitive course were detected. There was a significant improvement in global functioning as measured by the GAF-F over the follow-up period (*p* < 0.001), without any time x group interactions (*p* = 0.25). Participants with sustained negative symptoms had a significantly lower level of global functioning at 10-year follow-up with an additional independent effect of the cognitive composite score, compared to all other groups. Individuals with an early illness course characterized by absence of negative symptoms form a group with better cognitive and functional outcomes than the impairments typically associated with schizophrenia. Individuals with sustained levels of negative symptoms on the other hand may require a combined focus on both negative and cognitive symptoms.

## Introduction

Negative and cognitive symptoms are core features of schizophrenia (1–3). Both are consistently associated with poorer clinical and functional outcome (1, 4–8), yet currently few effective treatments for either exist (2). The suggestion of subtypes, such as type II schizophrenia (9), negative schizophrenia (10) or deficit schizophrenia (11) have highlighted the significant co-occurrence of negative and cognitive symptoms in a subset of patients. Although sustained negative symptoms is not currently seen as a marker of a distinct disease sub-group within the schizophrenia spectrum (12), the association between negative symptoms and cognition remains important. This association is present for a wide range of cognitive domains, including memory, processing speed, attention, and executive functions (13–16). Furthermore, both cognitive and negative symptom domains have been associated with early predictors of outcome suggesting possible targets for early intervention, prevention, and treatment planning (17, 18).

An important question regarding the relationship between negative and cognitive symptoms concerns their temporal relationship. Although negative symptoms are more stable than positive symptoms, longitudinal studies have shown that they do show variation over time (19–21). Using latent class analysis to identify symptom courses in a 10-year follow-up in the OPUS study, Austin et al. (21) identified four course types for negative symptoms (21). One group had symptoms that were consistently high, i.e., above moderate levels while another group showed only mild negative symptoms at baseline with no negative symptoms from 1 year until 10-year follow-up. The remaining two groups fell in-between and with more variability in symptom scores (21). It has been argued that distinguishing enduring high levels of negative symptoms from fluctuating negative symptoms or symptoms hovering around threshold levels is important, both from a theoretical and an empirical perspective (12, 22, 23). This distinction is also deserving of attention for clinical reasons, since the course of negative symptoms is related to functional outcome, especially social functioning, in individuals with schizophrenia (8, 24).

In a recent study, we used clinically meaningful cut-off values in a 1-year follow-up study (25) to investigate the longitudinal relationship between negative symptoms and cognitive functioning in first-episode psychosis (FEP) participants. The groups had either no (NNS), mild (MNS), transitory (TNS) or sustained (SNS) negative symptoms. We found a dose-effect type relationship between the level of negative symptoms and the level of cognitive functioning, with the largest group differences in cognitive functioning between FEP participants with SNS and NNS. The latter group did not differ significantly from healthy controls on any cognitive measure.

In the current study, our main aim was to follow-up these results and investigate how negative symptom severity over a longer (10-year) follow-up period was related to cognitive functioning in a group of FEP participants, using the same stratification as at 1-year follow-up but here based on baseline and 10-year symptom levels. As in our previous study we included a group of healthy controls to explore the relative cognitive performance of the four FEP groups, stratified for levels of negative symptoms, i.e., sustained, transitory, mild or no negative symptoms (25) over 10 years. To evaluate the clinical significance of any group differences, we added an assessment of global functioning as external validation. Based on our findings at 1-year follow-up we hypothesized that the main difference in cognitive functioning would be between the NNS and SNS groups. The specific research aims were as follows:

First, to investigate if the method of grouping participants according to negative symptoms at the 10-year follow-up would replicate the previous findings, i.e., would reproduce four groups of approximately the same size and with comparable clinical characteristics and differences in baseline cognitive functioning.

Second, to investigate the course of cognitive functioning over the follow-period, both in the entire sample and between groups.

Third, to investigate the course of global functioning over the follow-up period for the different negative symptom groups, and evaluate to what extent the putative difference between the negative symptom groups result from differences in cognitive functioning, in addition to the influence from other clinical symptoms.

## Methods

### Participants

Participants were recruited through the “Thematically Organized Psychosis” (TOP) study, an ongoing prospective cohort study recruiting participants from in- and outpatient clinics in the greater Oslo area and the Innlandet Hospital Trust region, in Norway. All FEP participants were assessed for the study within 1 year of starting their first adequate treatment for a psychotic episode (defined as hospital treatment in an acute/psychosis ward and/or antipsychotic medication in recommended dosage). For the current study, we included participants with broad schizophrenia spectrum disorder at baseline and at follow-up including the following DSM-IV diagnoses: schizophrenia, schizophreniform disorder, schizoaffective disorder, delusional disorder, brief psychotic disorder and psychosis NOS (12). Exclusion criteria were IQ below 70, not speaking a Scandinavian language, clinically significant head injury or age beyond the range 18–65.

**Figure 1:**
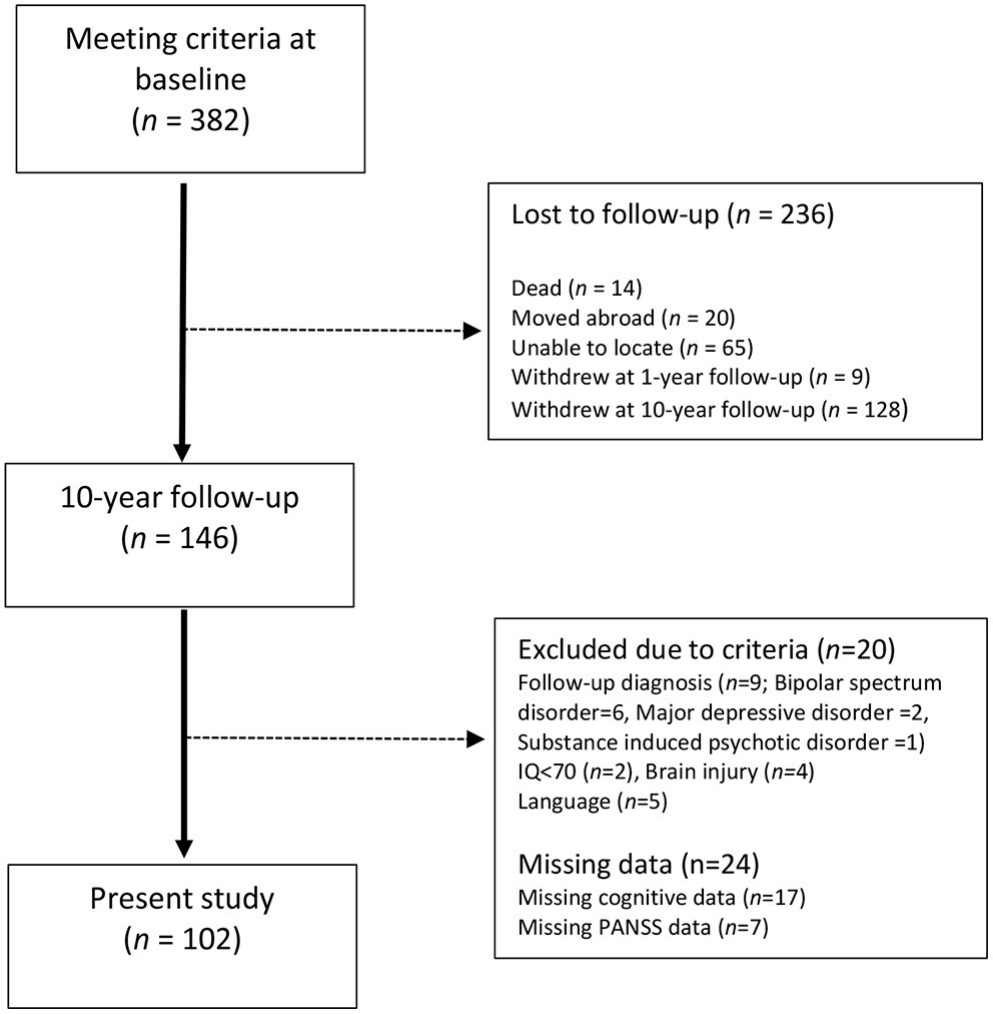
Account of attrition in the 10-year follow-up.

One hundred forty-six from a total of 382 participants with a broad schizophrenia spectrum diagnosis at baseline met for follow-up assessment at 10 years, a retention rate of 38.2%. A final sample of 102 participants who had undergone cognitive assessment at the 10-year follow-up met all criteria to participate in the current study. Figure 1 provides details about loss to follow-up and unmet inclusion criteria.

Healthy controls were recruited from the same catchment area as FEP participants and were invited by letter through random selection from the public population registry. They were screened using the Primary Care Evaluation of Mental Disorders (PRIME-MD) (26) to assess for symptoms of severe mental health disorders, and underwent a brief demographic interview at both baseline and 10-year follow-up including direct questions about mental disorders in the family. In addition to the listed exclusion criteria for FEP participants, healthy controls were excluded from the study if they met criteria for substance abuse or dependency in the last 6 months, or if they reported a history severe mental disorder in first-degree relatives. Only control participants who met for 1-year follow-up were contacted for 10-year follow-up. A total of 164 healthy controls from baseline were eligible for the 10-year follow-up study and a total of 120 met for reassessment, giving a retention rate of 73%. A total of 116 healthy controls completed necessary cognitive assessment at baseline and 10-year follow-up and were included and used to generate standardized scores on the cognitive tests. The resulting sample was significantly larger than any of the patient groups and had also different error variances. To meet the assumptions for conducting analyses of variance (ANOVAs) as the main statistical analyses, the sample was randomly reduced to 26 participants to fit the statistical purposes. Study participation required written informed consent using a form approved by the Regional Ethics Committee.

### Cognitive Assessment

A total of 14 tests were used to cover four cognitive domains which are known to be negatively influenced in schizophrenia: *Verbal learning and memory, attention, processing speed*, and *executive functions*. Cognitive assessments were carried out by psychologists or masters of neuroscience trained by senior research psychologists in the specific tests used and calibrated to ensure reliable test scores according to procedures developed at the research center. All test scores were converted into standardized Z-scores based on the total healthy control sample (*n* = 116).

The *verbal learning and memory* domain was comprised of trials 1–5 and delayed free recall from the California Verbal Learning Test (CVLT-II) (27), and the immediate and delayed recall conditions from the Logical Memory test in the Wechsler Memory Scale (WMS) (28). *Attention* was assessed using the Digit Span and Letter-Number Sequencing Test (28). *Processing speed* was assessed with the Digit Symbol Test (WAIS-III) (29), and the Color Naming and Word Reading subtest of the Color-Word Interference Test of the Delis-Kaplan Executive Function Scale (D-KEFS) (30). *Executive functions* were comprised of five separate scores from the D-KEFS test battery; the Inhibition and Inhibition/Switching subtests from the Color-Word Interference Test and Letter Fluency, Category Fluency and Category Switching from the Verbal Fluency Test. As a measure of general cognitive ability, a *cognitive composite score* was also calculated as the total sum of all test scores divided by the number of tests. *Current IQ* was measured with the abbreviated Wechsler intelligence scale WASI (31).

### Clinical Assessment

Clinicians with formal background as licensed psychologists, psychiatrists or psychiatric residents conducted diagnostic interviews at baseline and follow-up using the Structured Clinical Interview for DSM IV Axis I disorders (SCID-I) (32). All interviewers were trained according to a program developed at UCLA prior to conducting interviews, and the inter-rater reliability from this program has previously been evaluated and found satisfactory with overall agreement for DSM-IV diagnostic categories of 82% and an overall κ of 0.77 (95% CI:0.60–0.94) (33). The duration of untreated psychosis, time from onset of psychotic symptoms to first adequate treatment, was established based on information from interviews with participants and medical records. Symptoms were assessed with the Positive and Negative Syndrome Scale (PANSS) (34) using a validated five-factor model (35), but the depressive factor was excluded and instead measured by the Calgary Depression Scale for Schizophrenia (CDSS) (36) which is designed not to overlap with negative symptoms. Alcohol and substance use severity was assessed with the Alcohol Use Disorder Identification Test (AUDIT) and the Drug Use Disorder Identification Test (DUDIT) (37). The Global Assessment of Functioning Scale-Split version (GAF-F) (38) was used as a clinician rated measure of global functioning. The GAF-Split version assesses global symptoms and global functioning using two separate scales. The GAF-F thus serves the same purpose as the SOFAS.

### Negative Symptom Subgroup Definition

PANSS items N1, N2, N3, N4 and N6 have been recommended for studying negative symptoms because they measure core negative symptoms and do not conceptually overlap with cognition (1). The PANSS items are rated 1–7, where 1–2 is within what is considered normal variation, 3 is a mild symptom and 4 is clear symptomatology with increasing values up to 7 indicating a more severe pathology (34). The distinction between 3 and 4 is pivotal because a score of 3 marks the upper limit for widely used and validated remission criteria (39, 40), and a score of 4 is also commonly used as a lower limit in studies investigating negative symptoms (22, 23). Here, we followed the same approach and logic as in a previous 1-year follow-up study (25), but with negative symptoms at 10-year follow-up as the endpoint.

Four groups based on levels of negative symptom severity at two time-points were thus determined based on baseline and 10-year follow-up assessment:

1. Sustained negative symptoms (SNS): Participants with at least one item ≥ 4 at both time points.

2. Transitory negative symptoms (TNS): Participants with at least one item ≥ 4 at only one time point.

3. Mild negative symptoms (MNS): Participants with at least one item = 3 at one or both time points, but no item ≥ 4.

4. No negative symptoms (NNS): Participants with no item > 2 at either time point.

Based on these criteria, the SNS group consists of participants with enduring levels of clear negative symptomatology, while the NNS group consists of participants who did not exceed normal levels for any negative symptom item as assessed at the two time points. The MNS group presents with only mild symptomatology, while the TNS group presents with clear negative symptomatology at one time-point without the stability of the SNS group.

### Statistical Analyses

The Statistical Package for the Social Sciences version 27 (SPSS, Inc., Chicago, IL, USA) was used to perform all analyses.

Variables were inspected for normality and outliers. Due to skewness, the DUP variable was log-transformed before entering analyses, while median and range values were reported from the untransformed variable. ANOVA is sensitive to deviances in error variance when group size differences are large. Due to initial violations of assumptions made in analyses of variance (i.e., equality of covariance matrices and equality of error variances), the original group of healthy controls was randomly pruned from 116 to 26, the average size of the FEP groups (*n* = 102 divided into four groups; i.e., with an average size of ~26). Following this operation, all assumptions concerning sample size and error variances were met. Alpha level was set at 0.05 for all statistical tests. All reported *p-*values are two-tailed.

Analyses of variance (ANOVAs), chi-square test, and independent samples *t-*tests were used to compare groups for differences in demographic and clinical characteristics, including investigations of differences between participants retained and those lost to follow-up, as reported in Table 1. We used the following approach to identify and correct for putative confounding factors in the analyses of cognition: First, to be a potential confounder the variable in question had to show significant associations with both the negative symptom groups *and* with the assessment of cognitive functioning. Second, the variable in question did not have any criteria overlaps with (i.e., was not measuring parts of the same phenomenon as) either negative symptom group or cognitive functioning, since entering these would lead to spurious findings. Based on this, IQ, PANSS negative, and PANSS disorganized/concrete symptoms factor (41) were not included in any evaluations as putative confounders. For the remaining, we investigated associations between negative symptom-based groups, cognitive domains, and clinical characteristics using Spearman's rank correlations. The group variable was here treated as an ordinal scale based on putative severity, with healthy controls = 0, NNS = 1, MNS = 2, TNS = 3, and SNS = 4 (see Supplementary Table 1).

**Table 1:** Baseline descriptive information for the different patient groups.

**Variable**	**NNS**	**MNS**	**TNS**	**SNS**	***F/X^**2**^***	***df***	***P***
*N* 102 total (%)	18 (18)	31 (30)	36 (35)	17 (17)			
Age (yr)^a^	28.9 ± 8.4	26.6 ± 9.1	25.7 ± 8.2	24.1 ± 5.6	1.10	3	0.35
Women *N* (%)	9 (50)	15 (48)	19 (53)	5 (29)	2.68	3	0.44
Education (yr)^b^	13.5 ± 2.8	13.7 ± 3.3	12.5 ± 2.5	11.9 ± 2.4	2.23	3	0.09
IQ^c^	109.5 ± 12.9	105.4 ± 13.7	100.6 ± 13.6	97.6 ± 19.5	2.54	3	0.06
Age at onset (Psychosis)^d^	24.4 ± 8.7	22.7 ± 8.1	21.1 ± 6.8	21.6 ± 4.0	0.92	3	0.43
Duration of untreated psychosis, median (range)^e^	19.5 (780)	26 (1,299)	104 (1,039)	76 (774)	2.64	3	0.06
PANSS positive	9.3 ± 3.8	10.5 ± 3.5	11.8 ± 3.9	12.9 ± 4.8	3.18	3	**0.03**
PANSS disorganized	4.4 ± 1.9	5.1 ± 2.0	5.9 ± 2.2	8.1 ± 3.5	8.35	3	**<0.001**
PANSS excited^f^	5.6 ± 2.0	5.9 ± 1.9	6.2 ± 2.1	7.3 ± 3.1	2.00	3	0.12
AUDIT^g^	9.7 ± 7.9	7.1 ± 5.4	7.2 ± 8.0	6.5 ± 5.9	0.70	3	0.71
DUDIT^h^	6.1 ± 8.1	2.8 ± 6.4	1.4 ± 2.6	4.8 ± 6.9	3.00	3	**0.04**
Leverl of Antipsychotic medication in DDD	0.6 ± 0.6	0.7 ± 0.8	0.7 ± 0.6	0.9 ± 0.9	0.70	3	0.55
Antipsychotic medication yes/no	11/7	23/8	29/7	15/2	4.09	3	0.25
GAF-F	52.4 ± 16.5	46.8 ± 12.6	38.2 ± 9.3	37.5 ± 7.6	8.27	3	**<0.001**
Depression (CDSS total)^i^	4.7 ± 4.3	6.7 ± 4.5	9.4 ± 4.4	5.8 ± 3.5	5.69	3	**0.001**
Schizophrenia *N* (%)	7	13	22	13			
Schizophreniform *N* (%)	5	2	2	1			
Schizoaffective *N* (%)	1	4	6	1			
Psychosis NOS *N* (%)	5	7	6	2			
Delusional disorder (%)	-	4	1	-			
Brief psychotic disorder (%)	-	1	-	-			

*AUDIT, Alcohol Use Disorders Identification Test; CDSS, Calgary Depression Scale for Schizophrenia; DDD, defined daily dosage; DUDIT, Drug Use Disorders Identification Test; GAF-F, Global Assessment of Functioning-Functioning; IQ, intelligence quotient; MNS, mild negative symptoms; NOS, not otherwise specified; NNS, no negative symptoms; PANSS, Positive and Negative Syndrome Scale; SNS, sustained negative symptoms; TNS, transitory negative symptoms*.

a *When including the healthy controls age difference was significant F_4,123_ = 3.53, p = 0.009*.

b *Education years: number of missing scores: TNS = 2*.

c *When including the healthy controls IQ difference was significant F_4,123_ = 5.94, p < 0.001*.

d *Age at onset (Psychosis): number of missing scores: TNS = 2*.

e *Duration of untreated psychosis: missing data: MNS = 1, TNS = 1*.

f *PANSS excited: number of missing scores: MNS = 1*.

g *AUDIT: number of scores missing: NNS = 1, MNS = 3, TNS = 3*.

h *DUDIT: number of scores missing: MNS = 1, TNS = 3*.

i *CDSS: number of scores missing: NNS = 1, TNS = 2. P-values in bold are statistically significant (p > 0.05)*.

### Analysis of Differences in Baseline Cognitive Functioning

To investigate whether there was an overall difference in baseline cognitive functioning based on the level of negative symptoms over the follow-up period, we first performed a *multivariate analysis of variance (MANOVA)* as an omnibus test with “group” (the four negative symptom groups and healthy controls) as the independent variable and the four cognitive domains as dependent variables (Wilk's Λ). Further, given a significant group effect, follow-up explorations were done with separate ANOVAs for each cognitive domain and for the cognitive composite score, reporting partial η^2^ as effect size and *post-hoc* Bonferroni correction for multiple comparisons when relevant. There were no clinical symptoms that were statistically significantly associated with both negative symptoms-based groups and cognitive domains, and we thus did not proceed with ANCOVAs.

### Analysis of Differences in the Cognitive Course Between Groups

Differences between groups in the cognitive course over the 10-year study period were investigated by performing a series of repeated measures ANOVAs for each cognitive domain and the cognitive composite score (Pillai's Trace reported). As validation of the repeated measures ANOVAs, we performed additional linear mixed models for the five cognitive variables. Time (baseline vs. follow-up), group (HC and four negative symptom groups) and the time x group interaction were fixed. The models included a random intercept and were conducted with maximum likelihood estimation. The linear mixed models were undertaken with the complete HC sample (*n* = 116).

### Analysis of Global Functioning in Negative Symptom Groups

Our third aim was to investigate differences in global functioning between negative symptom groups and to assess the independent contributions of both group and cognition on GAF-F. We investigated group differences in GAF-F scores at baseline and follow-up using ANOVAs, and the development of GAF-F scores over time with separate repeated measures ANOVA (Pillai's Trace reported). Finally, we investigated the added contribution of cognitive functioning to global functioning using multiple linear regression analysis, with GAF-F at follow-up as the dependent variable and with the cognitive composite score and coming from the SNS group (vs. all other groups) as the two independent variables, corrected for differences in other clinical symptoms. Since the aim here was to identify the added contribution of cognition and not primarily to rule out confounder effects, the different symptom domains were entered independent of their association or lack of association to cognition. Symptom domains that did not have a significant contribution to the variation in functioning was not retained in the final model. Residual plots and evaluation of outliers were used to ascertain that the statistical requirements were met.

## Results

### Demographic and Clinical Characteristics

Participants lost to follow-up did not differ from those who met for 10-year assessment on IQ, age, gender, or any clinical measure at baseline. The age of the healthy control group (*M* = 32.7, *SD* = 7.9) and their IQ (M = 114.7, SD = 8.5) was statistically significantly higher than the clinical groups (*F*_4,123_ = 3.53, *p* =0.009) and (*F*_4,123_ = 5.94, *p* < 0.001). Clinical and demographic characteristics of the negative symptom groups are presented in Table 1. As expected, there were several significant between-group differences for clinical measures at baseline, including PANSS positive, depressive, and disorganized symptoms and GAF-F. As shown in Table 1, the negative symptom groups varied in size from 17 to 36 out of the total *N* = 102. As a proportion of the total, the NNS group was 18%, the MNS 30%, the TNS 35%, and the SNS 17%.

### Analysis of Differences in Baseline Cognitive Functioning

**Figure 2:**
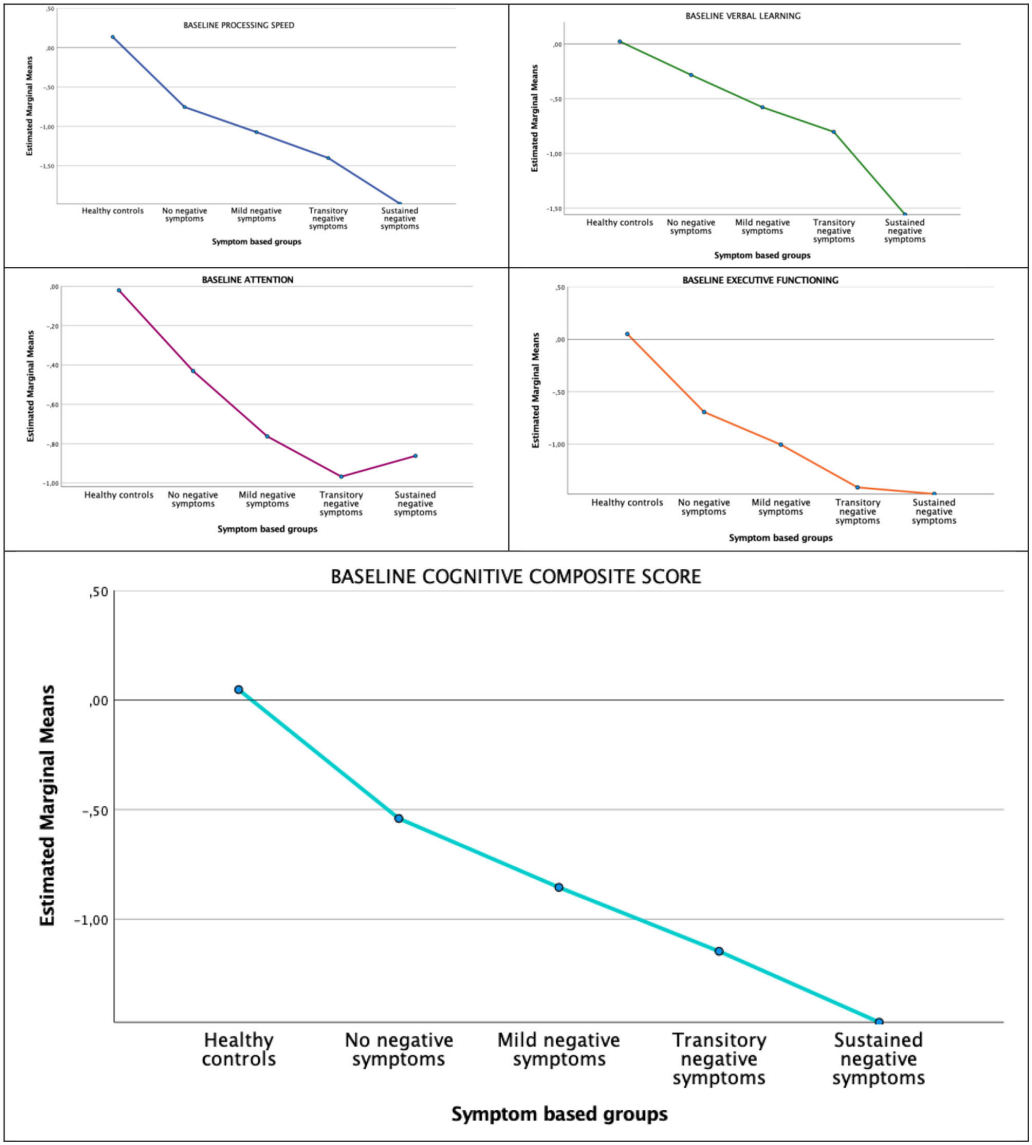
Cognitive domains by negative symptom groups at baseline.

Our first research question concerned differences in cognitive functioning at baseline. The main multivariate analysis (MANOVA) done to compare all five groups on overall baseline cognitive performance was significant, *F*_16,352_ = 3.18, *p* < 0.001; Wilk's Λ = 0.662, partial η^2^ = 0.10. The following separate ANOVAs for each cognitive domains are presented in Table 2 with a graphical illustration of the five groups according to cognitive domain and the cognitive composite score is presented in Figure 2. The observed differences between groups from the Bonferroni-corrected *post-hoc* analyses are given in Table 2. For any cognitive domain and for the cognitive composite score, the healthy controls did not differ significantly from the NNS group. The remaining negative symptom groups were outperformed by healthy controls on all domains and the cognitive composite score except for the MNS group on verbal learning and memory and the SNS group on attention. The NNS and the MNS group outperformed the SNS group on verbal learning and memory. The NNS group also differed significantly from the SNS group with superior performance on the cognitive composite. As displayed in Figure 2, the mean values for cognitive performance decreased stepwise with an increase in the burden of negative symptoms, and with the largest difference between the NNS and SNS groups. There were no differences in age between negative symptom groups, but since there was a significant age difference when including healthy controls, we conducted a follow-up analysis controlling for age. This analysis did not provide different results. Between-group differences in cognitive functioning at 10-year follow-up are presented in Supplementary Table 3.

**Table 2:** Baseline cognitive scores for the different patient groups and healthy controls.

	**NNS (18)**	**MNS (31)**	**TNS (36)**	**SNS (17)**	**HC (26)**	**ANOVA**			
	**Mean (SD)**	**Mean (SD)**	**Mean (SD)**	**Mean (SD)**	**Mean (SD)**	***F***	***P-*value**	**η^2^**	***Post-hoc* analysis**
Processing speed	−0.71 (1.1)	−1.05 (1.4)	−1.32 (1.4)	−1.98 (1.9)	0.14 (0.7)	7.88	*P* < 0.001	0.20	HC > MNS, TNS, SNS
Verbal learning and memory^a^	−0.28 (0.8)	−0.58 (1.1)	−0.80 (0.9)	−1.56 (1.2)	0.02 (0.7)	7.95	*P* < 0.001	0.21	HC > TNS, SNS|NNS>SNS|MNS>SNS
Attention^b^	−0.43 (1.0)	−0.76 (0.7)	−0.97 (0.9)	−0.86 (1.3)	−0.02 (0.9)	4.22	*P = 0.0*03	0.13	HC > MNS, TNS
Executive functioning	−0.70 (1.1)	−0.97 (1.1)	−1.30 (1.2)	−1.48 (1.4)	0.05 (0.7)	7.55	*P* < 0.001	0.20	HC > MNS, TNS, SNS
Cognitive composite	−0.54 (0.8)	−0.83 (0.8)	−1.06 (0.9)	−1.47 (1.2)	0.05 (0.6)	9.75	*P* < 0.001	0.24	HC > MNS, TNS, SNS|NNS>SNS

*ANOVA, analysis of variance; NNS, No negative symptoms; MNS, Mild negative symptoms; TNS, Transitory negative symptoms; SNS, Sustained negative symptoms; HC, Healthy contols*.

a, b *NNS (n = 16), MNS (n = 30) and TNS (n = 34) due to missing data*.

### Analysis of Differences in the Cognitive Course Between Groups

**Figure 3:**
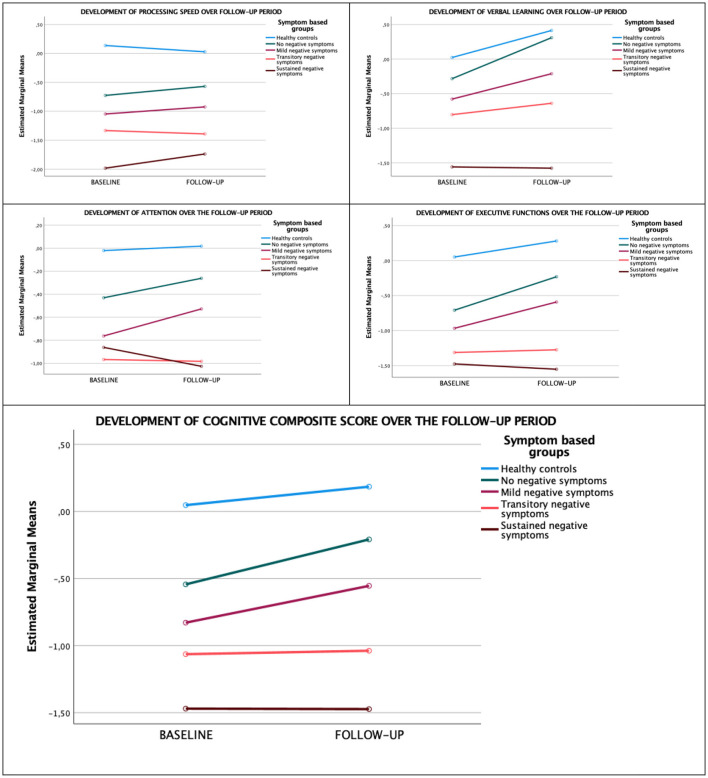
Development of cognitive domains over the 10-year follow-up.

*Between-group* differences in cognitive course over the 10-year follow-up for each domain and the cognitive composite score are displayed in Figure 3. There was a significant improvement in verbal learning (F_1,118_ = 17.87, *p* < 0.001; Pillai's Trace = 0.132), executive functioning (F_1,121_ = 4.86, *p* = 0.03; Pillai's Trace = 0.039), and the cognitive composite (F_1,123_ = 8.34, *p* = 0.005; Pillai's Trace = 0.063) over time. Visual inspection of means plots suggested that these improvements were mainly due to the healthy controls, the NNS and MNS groups, but there were no significant time x group interaction effects. The linear mixed model analyses, which included the complete HC sample, confirmed these results. All five analyses yielded significant effects of group. In addition, the effects of time were significant for verbal learning [*b* = 0.28, *t*(213.61) = 3.28, *p* = 0.001], executive function [*b* = 0.21, *t*(217.79) = 2.05, *p* = 0.041], and the composite score [*b* = 0.16, *t*(218.00) = 2.53, *p* = 0.012]. None of the interaction effects were significant.

### Analysis of Global Functioning in Negative Symptom Groups

**Figure 4:**
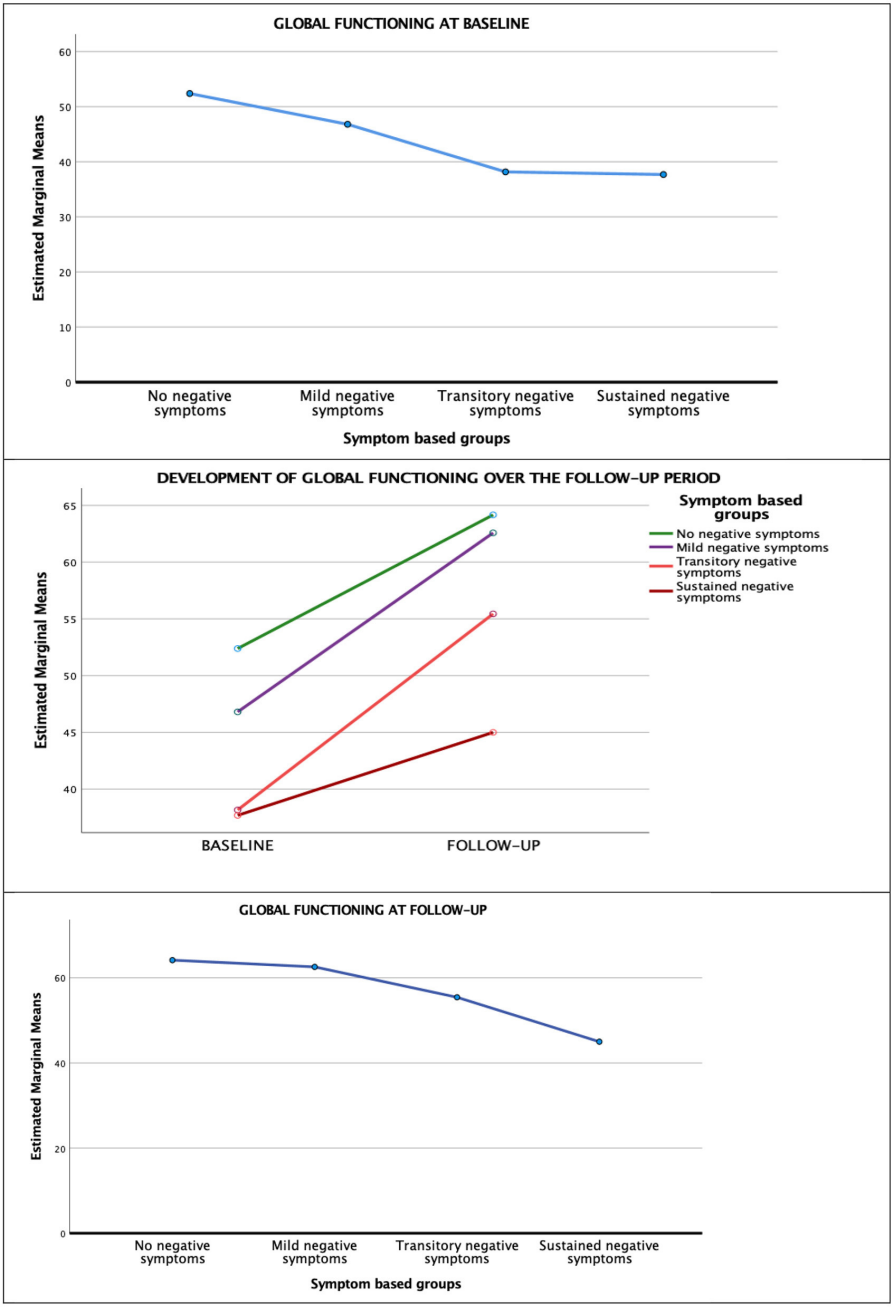
Global functioning (gaf-f) over the 10-year follow-up.

Global functioning at baseline, follow-up, and in the course over the 10-year follow-up is displayed in Figure 4. There was a significant improvement in global functioning as measured by the GAF-F over the follow-up period (F_1,97_ = 49.97, *p* < 0.001; Pillai's Trace = 0.340), without any time x group interactions (F_1,97_ = 1.40, *p* = 0.25; Pillai's Trace = 0.041). There were significant between-group differences in GAF-F at follow-up. The SNS group had the poorest level of global functioning, and the largest differences in GAF-F score were between this group and the NNS (mean difference 13.34, *p* = 0.018) and the MNS (mean difference 13.35, *p* = 0.007) groups. To explore the potential added contribution of cognitive functioning to the groups-based differences in global functioning, we performed a multiple linear regression analysis with GAF-F as the dependent variable and with the cognitive composite score and coming from the SNS group (vs. all other groups) as the independents, correcting for differences in other symptom areas, Both the cognitive composite score (beta = 0.98, *p* < 0.001), the PANSS positive component score (beta = 0.28, *p* < *0.001*) and the PANSS depressive component score (beta 0.30, *p* < *0.001*) had significant contributions to the variation in GAF-F at follow-up. Still, belonging to the SNS group had an independent and statically significant contribution to GAF-F when entered at the last step of the analysis (beta = 3.4, *p* = *0.038*) (adjusted model R^2^ = 0.49, F = 42.6, *p* < *0.001*).

## Discussion

Using baseline and 10-year follow-up data to group FEP patients based on negative symptom severity, the current study largely replicates findings from our previous 1-year follow-up study, albeit with minor variations in group size (25). The SNS group which comprised 17% of the FEP sample is approximately the same size as reported for groups with deficit schizophrenia (42) or persistent negative symptoms (43) elsewhere. We also replicate our previous finding of group differences in cognitive functioning at baseline between the four groups. The *post-hoc* analyses showed that the NNS group was not significantly outperformed by healthy controls on any domain or on the cognitive composite score. With some minor exceptions the healthy controls outperformed the remaining negative symptom groups on the four cognitive domains and on the cognitive composite. In the FEP sample, the NNS group significantly outperformed the SNS group on the verbal learning and memory domain and on the cognitive composite score. Our findings indicate that we already after 1 year of treatment in FEP can identify valid subgroups based on stratification of negative symptoms that have relevance for long-term outcome.

Concerning our second aim, we found parallel courses in the cognitive domains across subgroups. Significant improvements were detected for the cognitive composite and the domains of verbal learning and memory and executive function. The domains of processing speed and attention remained stable. These results add to the mixed findings in FEP, suggesting mainly stability (44), but improvements have also been reported (45). A recent 10-year follow-up study with healthy controls and a similar FEP sample size as the present, also reported a generally stable cognitive course (46). Although the authors reported finding a subgroup with a deteriorating course, they were unable to identify significant predictors. Upon visual inspection of slopes the SNS group did not show the same tendency toward improvement as the other groups, but there were no statistically significant interaction effects that would indicate a difference in course.

Concerning our third aim, we found that the groups differed significantly in their level of global functioning at baseline but did not differ in their course of global functioning over the 10-year follow-up period. The main difference in global functioning was as expected between the NNS and the SNS groups. However, the SSN group mainly showed a stable course and not clear deterioration. This is in line with some (47, 48), but not all (49–51), studies of the development of functioning over time in FEP. We also found that the differences in cognitive functioning had an independent contribution to global functioning, beyond the effect of severe negative symptoms as represented by belonging to the SNS group. Since the addition of a measure of functioning was added to serve as an external validator of clinical relevance, we conducted analyses of global functioning and the cognitive composite score not including functional- and/or cognitive subdomains. The latter type of analyses could give a more in-depth understanding of the relationship between cognition and functioning but was outside the focus of the current paper which was the relationship between negative symptoms and cognition.

In addition to the significant and long-term effect of sustained negative symptoms, the most interesting finding in the current study is that the NNS group did not differ significantly from healthy controls for any cognitive measure. This replicates our previous findings from the 1-year follow-up of the current group and strengthens our argument that comparting the extreme groups of patients with stable absence or presence of negative symptoms would enhance our ability to explore the relationships between cognitive and negative symptoms. Future research would profit from more theory-driven approaches to the study of negative symptoms.

Moreover, our findings add to the broader discussion of the defining features of schizophrenia, as they show that a stable absence of negative symptoms is linked to more subtle deficits in cognition and less functional impairment. As noted by a recent review, there is a growing literature questioning the emphasis on positive symptoms to define the diagnostic category of schizophrenia (52). According to this view, both negative and cognitive symptoms are more specific to schizophrenia than positive symptoms, in line with the former Bleulerian concept of the disorder (52). In the absence of clear biomarkers, the “correct” diagnostic criteria remain elusive. Our findings do, however, suggest that characteristics important to the original concept of schizophrenia (i.e., cognitive, and functional impairments) are more closely associated with negative symptoms, rather than the positive symptoms often used to define the diagnosis. This is an additional argument for more in-depth studies of negative syndromes and their neuroscientific underpinnings.

### Future Research

Contrasting individuals with NNS and SNS has the potential to give new insights into negative symptoms, their association with cognitive symptoms, and relevant biomarkers including genetics and brain phenotypes captured by imaging techniques. In addition, more specific and elaborate measures of functional domains and particularly real-world functioning would also add to our understanding of their functional consequences. Furthermore, including frequent measurement points based on smart phone technology could provide more detailed data on the course of negative symptoms in critical periods of their developments. Finally, our study was planned before general access to good and reproducible measures of social cognition and thus did not include any such assessments at baseline.

### Strengths and Limitations

The strengths of this study are the longitudinal design, and the inclusion of a sample recruited through the Norwegian public health system, which covers all citizens regardless of socioeconomic status. The sample is also well-characterized, with validated assessments for both clinical and cognitive variables.

A clear limitation is a 10-year period without any in-between measurements that could map variability in negative symptoms. However, previous studies have indicated considerable stability in negative symptoms from 1- to 10-year follow-up (53).

Also, the loss of participants from baseline to follow-up is always a threat to the representativeness of the sample, and the retention rate in this study is low. However, a study simulating the effect of losing participants in long-term longitudinal studies found that association between variables was not affected even with high rates of attrition (54). Moreover, there were no significant differences in baseline demographic and clinical variables between participants included compared to those lost to follow-up in the current study. We are, however, not able to rule out attrition bias due to different courses of illness.

The statistical tests used do not make assumptions about the equality of sample sizes, and type I errors are not increased by this limitation. However, there might be an increase of type II error due to the small and unequal sample sizes, particularly concerning the main groups of interest, the NNS and SNS groups, since they were the smallest. This could cause a conservative bias in the statistical interpretation overlooking group differences that in fact are present.

Finally, our study was planned before general access to good and reproducible measures of social cognition and thus did not include any such assessments at baseline. This can be considered a limitation as social cognition has been found to mediate the relationship between neurocognition and functioning (55).

## Conclusion

Stratifying FEP patients based on the severity of negative symptoms over time could be key to understanding important aspects of heterogeneity in schizophrenia, such as the differences in cognitive functioning. This particularly applies to the differences between patients with persistently absent and persistently present negative symptoms. In the current study, participants with persistently low levels of negative symptoms over the 10-year follow-up period did not differ significantly from healthy controls and largely outperformed participants with sustained moderate-severe negative symptoms on verbal learning and memory. The group with persistent negative symptoms also demonstrated inferior global functioning, with an additional independent contribution from the difference in cognitive functioning. Clinical implications of the study are that differences in course of negative symptoms may indicate different treatment needs, and that the SNS group may need interventions specifically targeting cognitive impairments such as cognitive remediation. Although cognitive remediation does not primarily target negative symptoms, several studies have shown that in addition to improving cognition (56) this intervention may also have a beneficial effect on negative symptoms (57).

## Data Availability Statement

The raw data supporting the conclusions of this article will be made available by the authors, without undue reservation.

## Ethics Statement

The studies involving human participants were reviewed and approved by Regional Etisk Komite Sør Øst. The patients/participants provided their written informed consent to participate in this study.

## Author Contributions

TU, CS, AV, and ME formed the idea and planned the study. ME wrote the first draft and did the initial data analyses. IM contributed with data analysis and making of graphical presentation. GÅ, KW, BH, LW, CF, SL, and AF contributed with data collection. All authors have commented and contributed to the final submitted text.

## Funding

The study was supported by grants from the Research Council of Norway to NORMENT CoE (grant number c, under the Centers of Excellence funding scheme), Stiftelsen Kristian Gerhard Jebsen (SKGJ-MED-008), the Southern and Eastern Norway Regional Health Authority (#2006233, #2006258, #2011085, #2014102, #2015088), and the Research Council of Norway (#287714). The funding bodies had no role in the analyses or writing of the manuscript, or the decision to submit this work for publication.

## Conflict of Interest

The authors declare that the research was conducted in the absence of any commercial or financial relationships that could be construed as a potential conflict of interest.

## Publisher's Note

All claims expressed in this article are solely those of the authors and do not necessarily represent those of their affiliated organizations, or those of the publisher, the editors and the reviewers. Any product that may be evaluated in this article, or claim that may be made by its manufacturer, is not guaranteed or endorsed by the publisher.
