# Supplementary material for: Cognitive and Global Functioning in Patients With First-Episode Psychosis Stratified by Level of Negative Symptoms. A 10-Year Follow-Up Study
Source: Front Psychiatry. 2022 Mar 25;13:841057. doi: 10.3389/fpsyt.2022.841057 (PMC8990888; doi:10.3389/fpsyt.2022.841057)
Supplement: Supplementary file 1 [file Data_Sheet_1.pdf]

Supplementary material:

Table S1: Bivariate correlations (Spearman's rho) between clinical characteristics and cognitive domains at baseline and follow-up.

	Symptom groups	BL_Processing_Speed	BL_Verbal_Learning	BL_Attention	BL_Executive	BL_Composite
BL_Processing_Speed	-0,463**					
BL_Verbal_Learning	-0,431**	0,595**				
BL_Attention	-0,355**	0,481**	0,505**			
BL_Executive	-0,445**	0,861**	0,594**	0,569**		
BL_Composite	-0,495**	0,901**	0,774**	0,715**	0,927**	
BL_Positive	0,261**	-0,189	-0,137	-0,084	-0,054	-0,147
BL_Excited	0,179	0,062	-0,066	0,116	0,058	0,06
BL_Depressive	0,062	0,021	0,087	0,035	-0,058	0,002
BL_audit_score	-0,136	-0,031	0,071	0,022	0,063	0,036
BL_dudit_score	0,034	-0,03	-0,049	0,051	-0,028	-0,017
BL_GAF_function	-0,422**	00,371**	00,301**	00,272**	00,344**	00,396**
	**Symptom groups**	**FU_Processing_Speed**	**FU_Verbal_Learning**	**FU_Attention**	**FU_Executive**	**FU_Composite**
FU_Processing_Speed	-0,424**					
FU_Verbal_Learning	-0,519**	0,554**				
FU_Attention	-0,374**	0,514**	0,446**			
FU_Executive	-0,440**	0,824**	0,519**	0,653**		
FU_Composite	-0,537**	0,884**	0,734**	0,762**	0,912**	
FU_audit_score	-0,210*	0,049	0,122	0,132	0,094	0,117
FU_dudit_score	0,153	-0,023	-0,115	0,059	0,003	-0,005
FU_Positive	0,184	-0,262**	-0,246*	-0,158	-0,212*	-0,254**
FU_Excited	0,074	-0,019	-0,073	-0,006	-0,091	-0,049
FU_Depressive	0,16	-0,112	-0,135	-0,147	-0,121	-0,143
FU_GAF_function	-0,411**	0,309**	0,370**	0,306**	0,350**	0,391**

* p< 0.05; ** p< 0,01

Table S2. Follow-up descriptive information for the different patient groups							
**Variable**	**NNS**	**MNS**	**TNS**	**SNS**	***F/X²***	***df***	***P***
PANSS positive	7.8 ± 4.2	6.6 ± 2.7	8.0 ± 3.9	9.9 ± 4.9	2.87	3	**.04**
PANSS disorganized	4.1 ± 1.6	3.8 ± 1.6	4.6 ± 2.0	6.3 ± 2.9	5.79	3	**.001**
PANSS excited	4.9 ± 1.8	4.4 ± 0.7	4.8 ± 1.3	4.9 ± 1.8	1.11	3	.35
AUDIT	7.3 ± 7.1	5.6 ± 5.0	4.6 ± 6.8	6.9 ± 7.7	0.71	3	.59
DUDIT	2.5 ± 6.2	0.7 ± 2.0	0.5 ± 1.1	2.4 ± 3.6	2.68	3	**.04**
GAF-F	64.1 ± 16.9	62.6 ± 13.5	55.4 ± 17.7	45.0 ± 13.2	5.76	3	**.001**
Depression (CDSS total)	2.3 ± 3.1	2.7 ± 3.4	3.4 ± 2.9	2.9 ± 3.5	.413	3	.74
Follow-up time	9.5 ± 1.2	9.5 ± 1.6	8.8 ± 1.7	8.7 ± 1.7	1.9	3	.10

AUDIT=Alcohol Use Disorders Identification Test; CDSS=Calgary Depression Scale for Schizophrenia; DUDIT=Drug Use Disorders Identification Test; GAF-F=Global Assessment of Functioning-Functioning; MNS=mild negative symptoms; NOS=not otherwise specified; NNS=no negative symptoms; PANSS=Positive and Negative Syndrome Scale; SNS=sustained negative symptoms; TNS=transitory negative symptoms

Table S3. 10-year follow-up cognitive scores for the different patient groups and healthy controls.									
	NNS (18)	MNS (31)	TNS (36)	SNS (17)	HC (26)	ANOVA			
	Mean (SD)	Mean (SD)	Mean (SD)	Mean (SD)	Mean (SD)	*F*	*p*-value	η²	*Post hoc* analysis
Processing speed	-0,57 (0.9)	-0,92 (1.3)	-1,39 (1.6)	-1,7 (1.9)	0.03 (0.8)	6.16	*p*<.001	.17	HC>TNS, SNS
Verbal learning and memory[a]	0,28 (0.8)	-0,20 (1.0)	-0,61 (0.9)	-1,58 (1.2)	0.41 (0.7)	13.65	*p*<.001	.31	HC>TNS, SNS│NNS>SNS, TNS│MNS>SNS
Attention[b]	-0,26 (0.9)	-0,50 (0.9)	-0,91 (1.1)	-1,03 (1.0)	-0.02 (0.9)	4.92	*p*=.001	.14	HC>SNS, TNS
Executive functioning[c]	-0,23 (0.8)	-0,59 (1.1)	-1,27 (1.6)	-1,55 (1.7)	0.28 (0.9)	8.36	*p*<.001	.22	HC>TNS, SNS│NNS>SNS
Cognitive composite	-0,21 (0.7)	-0,55 (0.8)	-1,04 (1.1)	-1,47 (1.2)	0.19 (0.6)	11.86	*p*<.001	.28	HC>MNS, TNS, SNS│NNS>SNS,TNS│MNS>SNS

ANOVA = analysis of variance; NNS = No negative symptoms; MNS = Mild negative symptoms; TNS = Transitory negative symptoms; SNS = Sustained negative symptoms; HC = Healthy controls.

FIGURE S1: COGNITIVE DOMAINS BY SYMPTOM GROUPS AT FOLLOW-UP.

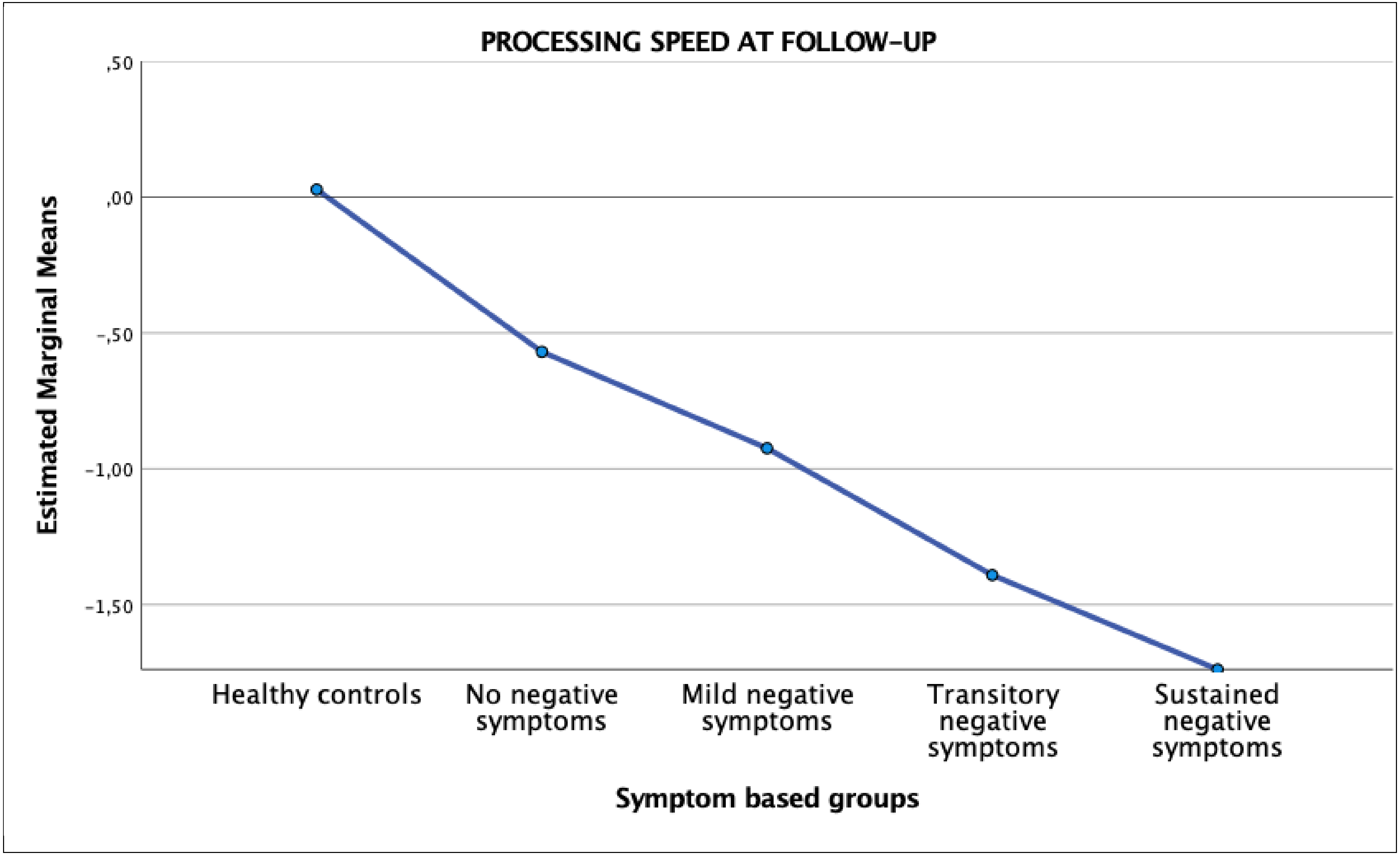

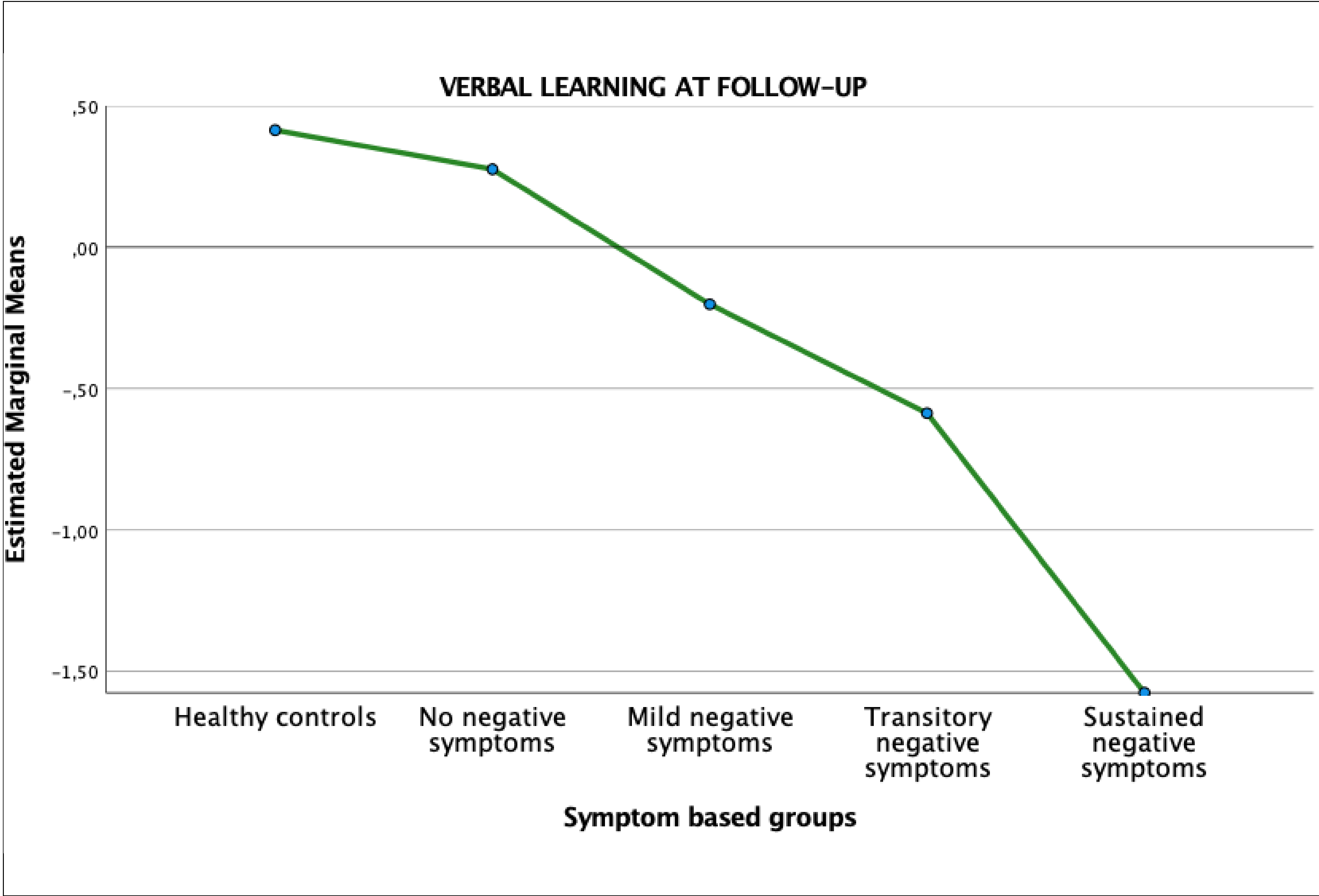

VERBAL LEARNING AT FOLLOW-UP
,50
,00
-,50
-1,00
-1,50
Estimated Marginal Means
Healthy controls
No negative symptoms
Mild negative symptoms
Transitory negative symptoms
Sustained negative symptoms
Symptom based groups

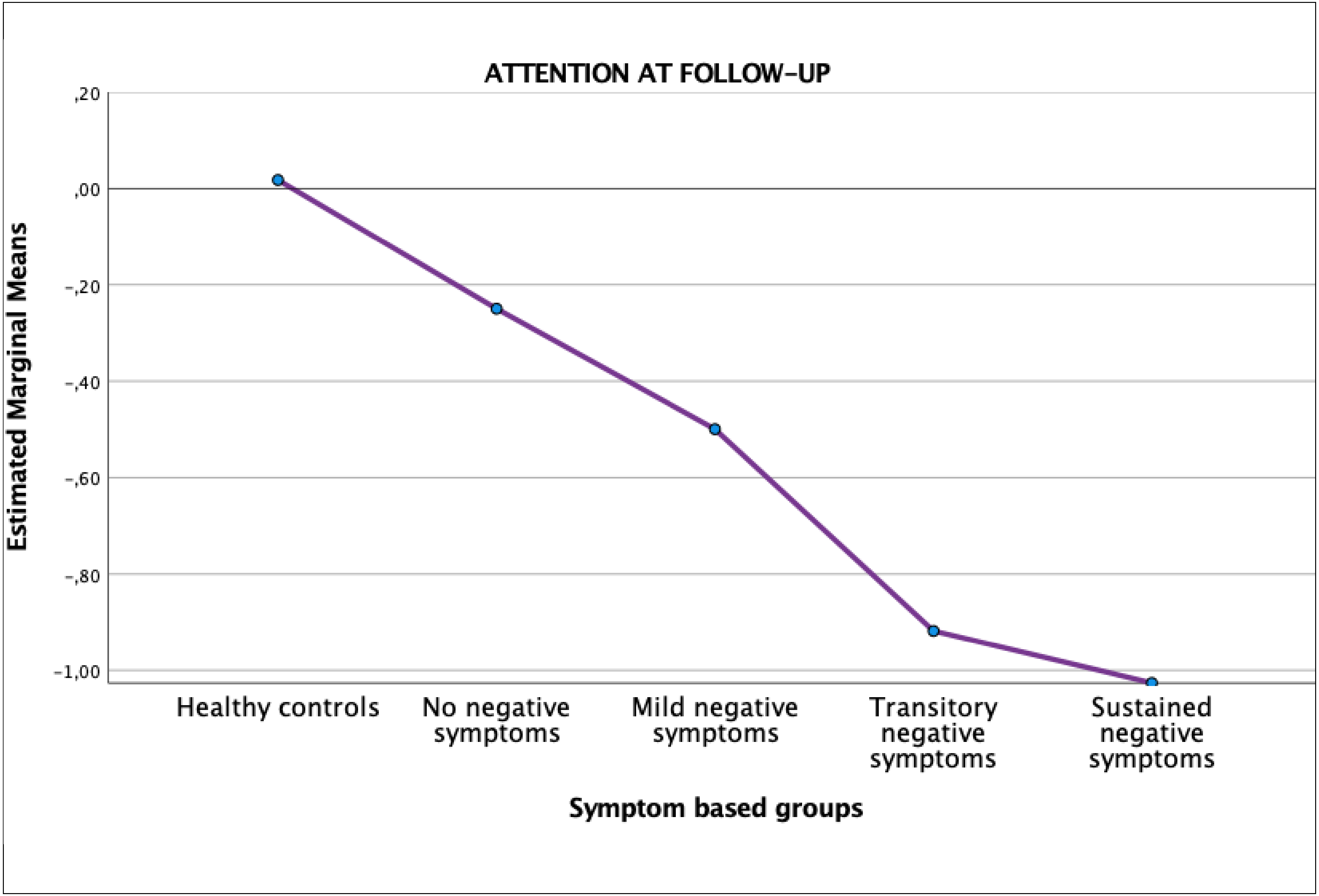
ATTENTION AT FOLLOW-UP
Estimated Marginal Means
,20
,00
-,20
-,40
-,60
-,80
-1,00
Healthy controls
No negative symptoms
Mild negative symptoms
Transitory negative symptoms
Sustained negative symptoms
Symptom based groups

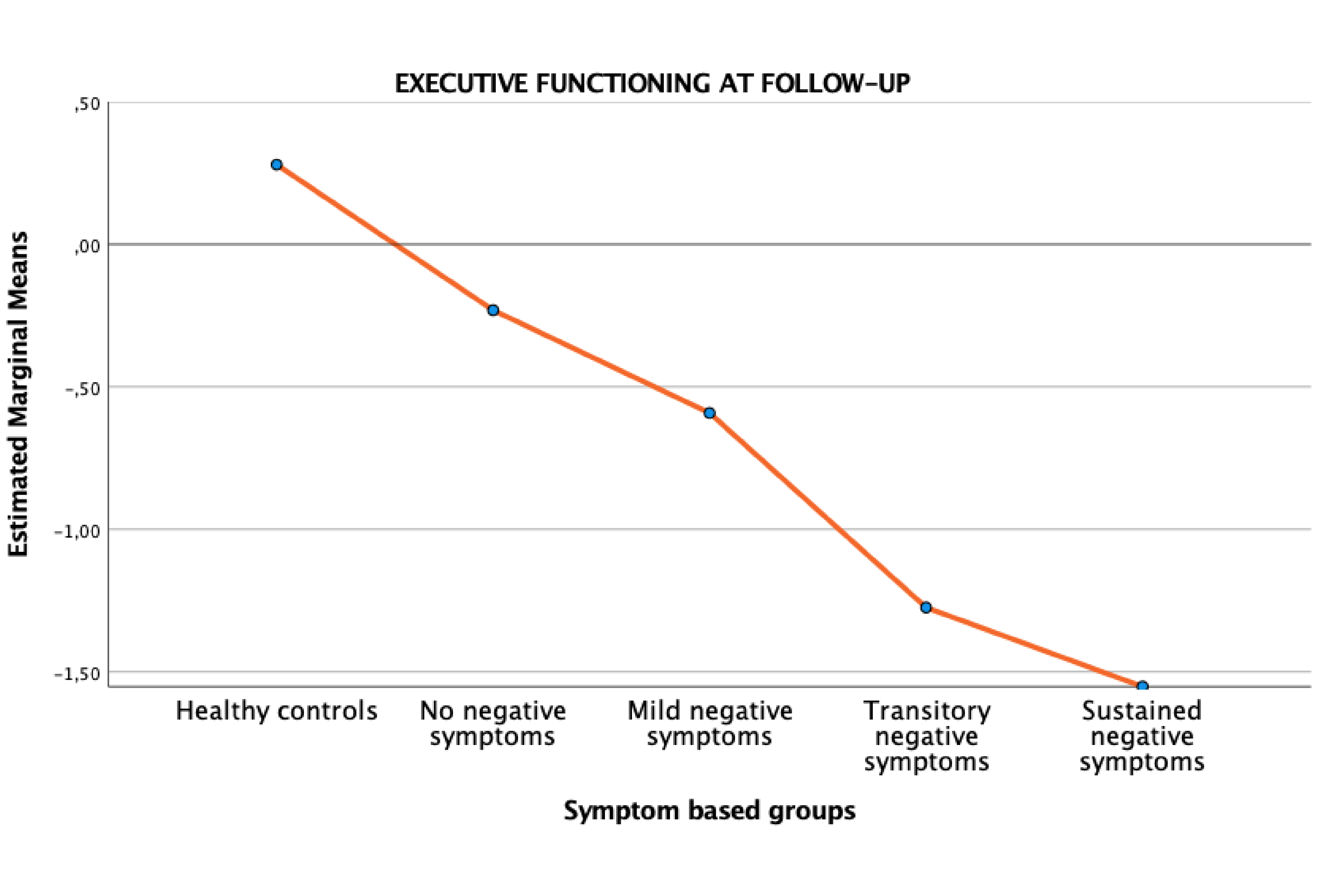
EXECUTIVE FUNCTIONING AT FOLLOW-UP
Estimated Marginal Means
,50
,00
-,50
-1,00
-1,50
Healthy controls
No negative symptoms
Mild negative symptoms
Transitory negative symptoms
Sustained negative symptoms
Symptom based groups

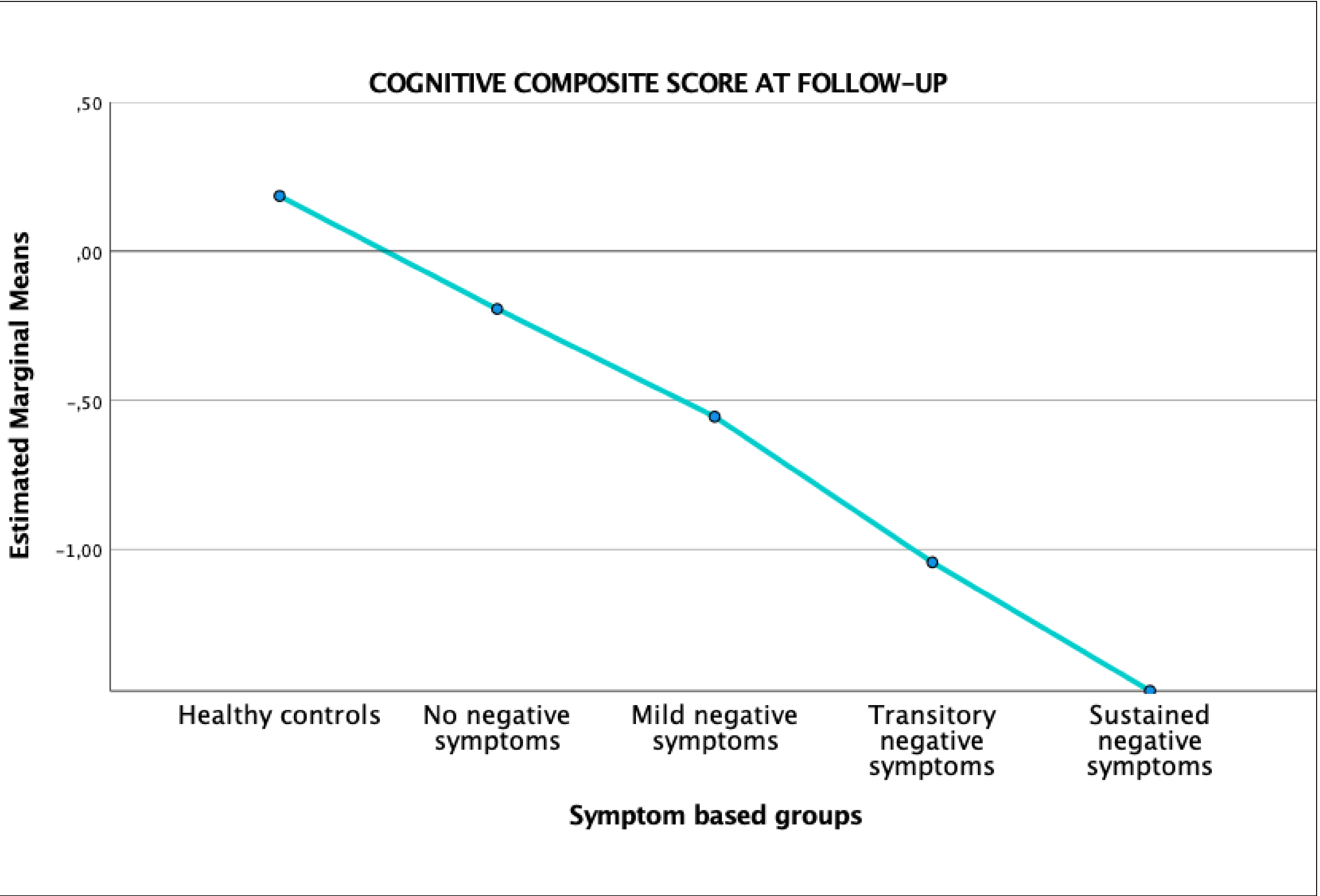

COGNITIVE COMPOSITE SCORE AT FOLLOW-UP
,50
,00
-,50
-1,00
Estimated Marginal Means
Healthy controls
No negative symptoms
Mild negative symptoms
Transitory negative symptoms
Sustained negative symptoms
Symptom based groups
